# Probing emission of a DNA-stabilized silver nanocluster from the sub-nanosecond to millisecond timescale in a single measurement†

**DOI:** 10.1039/d2sc01137a

**Published:** 2022-04-21

**Authors:** Mikkel Baldtzer Liisberg, Stefan Krause, Cecilia Cerretani, Tom Vosch

**Affiliations:** Nanoscience Center, Department of Chemistry, University of Copenhagen Universitetsparken 5 2100 Copenhagen Denmark tom@chem.ku.dk

## Abstract

A method for measuring emission over a range of sub-nanosecond to millisecond timescales is presented and demonstrated for a DNA-stabilized silver nanocluster (DNA-AgNC) displaying dual emission. This approach allows one to disentangle the temporal evolution of the two spectrally overlapping signals and to determine both the nano- and microsecond decay times of the two emission components, together with the time they take to reach the steady-state equilibrium. Addition of a second near-infrared laser, synchronized with a fixed delay, enables simultaneous characterization of optically activated delayed fluorescence (OADF). For this particular DNA-AgNC, we demonstrate that the microsecond decay times of the luminescent state and the OADF-responsible state are similar, indicating that the OADF process starts from the luminescent state.

## Abstract 2

A method for measuring emission over a range of sub-nanosecond to millisecond timescales is presented and demonstrated for a DNA-stabilized silver nanocluster (DNA-AgNC) displaying dual emission.

## Graphical abstract

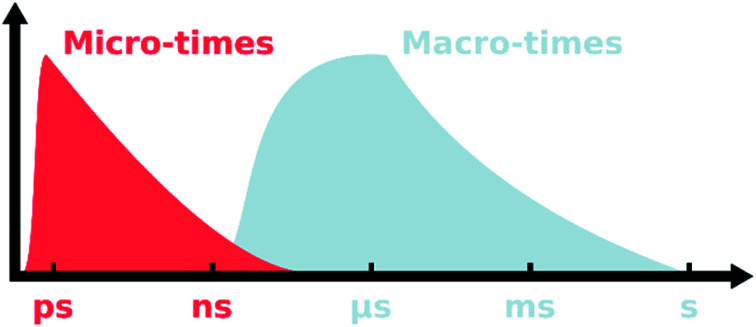

## Introduction

DNA-stabilized silver nanoclusters (DNA-AgNCs) are a class of emitters with intriguing photophysics dictated by the protecting DNA scaffold.^1–5^ Depending on the DNA sequence, DNA-AgNCs with vastly different spectroscopic properties can be obtained. As such, DNA-AgNCs, with emission spanning the visible^6,7^ and near-infrared (NIR)^8,9^ range, have been prepared with decay times in the nano-^10^ and microsecond regimes.^11,12^ While nanosecond decay times have been reported for DNA-AgNCs using time-correlated single photon counting (TCSPC), longer lived microsecond luminescence has only been very recently reported using pulsed Xe flash lamps or burst mode measurements.^11–13^ DNA-AgNCs have long been known to possess microsecond lived dark states, which manifest themselves as fluorescence blinking in single molecule and fluorescence correlation experiments.^14–16^ Considered to be non-emissive, these dark states have been utilized to generate background-free imaging capabilities by modulating the fluorescence intensity output or by exploiting a process called optically activated delayed fluorescence (OADF).^17–19^ OADF is similar to thermally activated delayed fluoresce (TADF),^20^ but instead of thermal repopulation, a second NIR excitation source is used to repopulate the fluorescent state. Time gating allows one to extract the OADF signal and generate a background free Anti-Stokes signal.^17^ The recent discovery of microsecond emission from DNA-AgNCs,^11,12^ led us to wonder whether these states are also able to generate OADF.

Since some DNA-AgNCs seem to emit photons over a large time domain, a single experiment covering this entire range will simplify characterization and provide the needed information to create a more complete picture of the excited state processes. The concept of simultaneously determining fluorescence and phosphorescence has previously been implemented for lifetime imaging (FLIM/PLIM) with a confocal microscope and a TCSPC counting module.^21,22^ In this application, a micro- to millisecond burst of high repetition rate excitation light (which enables to determine the nanosecond lived fluorescence) builds up a population of microsecond lived states that, after turning off the excitation source, allows any long-lived luminescence to fully decay without interfering fluorescence. This cycle is repeated until sufficient photons are collected for constructing both the nano- and microsecond decay curves. The burst approach is a convenient way to build up a population of species with a decay time that is longer than the pulse repetition time.^23,24^

We present here a similar and generally applicable method for studying systems exhibiting both nano- and microsecond decay times. As an exemplary system, we studied DNA-Ag_16_NC,^25^ which shows both nanosecond and microsecond emission at room-temperature (RT).^13^ The pulsed burst mode measurements were combined with secondary NIR excitation to elucidate the origin of the state responsible for OADF. By using time gating schemes, we were able to extract the decay time of the state responsible for OADF, which was similar to the decay time of the microsecond lived luminescent state.

## Results and discussion

### Simultaneous detection of ns fluorescence and μs luminescence

**Fig. 1:**
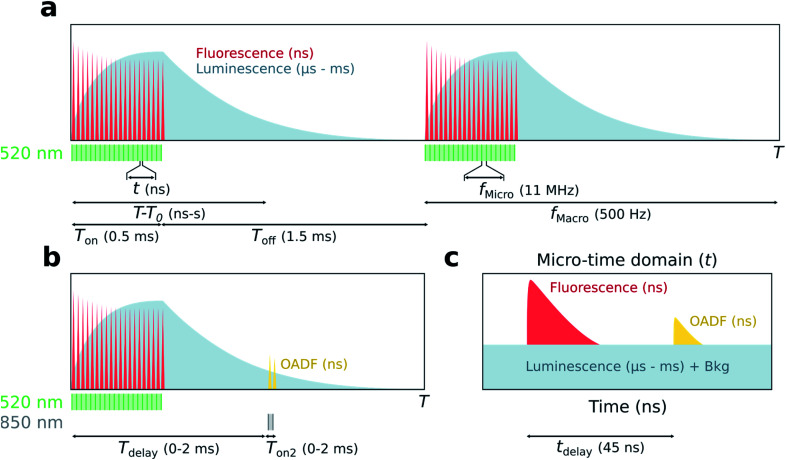
Principle of simultaneous detection of fluorescence and luminescence from micro- and macro-times. (a) In the macro-time domain, photon arrival times (*T*–*T*_0_) are recorded with a time resolution of 50 ns from an internal timer. A luminescent population is built up during the on period (*T*_on_) of the excitation source (520 nm), while the decay of the luminescent state is detected during the off period (*T*_off_). (b) The macro-time dynamics of OADF are probed by co-illuminating with a secondary excitation source (850 nm) of variable delay (*T*_delay_) and on period (*T*_on2_). (c) The micro-time domain represents the photon arrival times compared to the sync (*t*) and contains fluorescence decay information with sub-ns time resolution; μs luminescence will appear as a constant background together with detector dark counts and after pulsing (Bkg). The temporal dynamics of OADF can be studied by adding a secondary excitation source with delay, *t*_delay_.

The principle of simultaneous fluorescence and luminescence decay determination by TCSPC is depicted in Fig. 1 and the setup used is described in Fig. S1.† The method relies on assigning a ‘macro-time’ and a ‘micro-time’ to each detected photon. The macro-time (*T*–*T*_0_) is the arrival time of a photon from the start of the experiment (*T*_0_) to the detection event (*T*) and has a time resolution of 50 ns from an internal timer. Note that this time is actually the time of the synchronization signal (sync signal) of the next laser pulse and not the actual photon detection event. The micro-time (*t*) is the photon arrival time compared to the next sync signal and has a sub-ns time resolution.

The assignment of a micro- and macro-time to every photon brings many advantages. Histogramming the micro-times yields the nanosecond fluorescence decay curve, while long lived luminescence will appear as an increased background in the micro-time domain (Fig. 1c). To determine the luminescence decay (Fig. 1a), the high repetition rate of the 520 nm excitation source (*f*_Micro_ = 11 MHz) is kept on for a certain period (*T*_on_) to build up a population of the luminescent state, followed by a period where the excitation source is turned off until the next burst cycle starts (*T*_off_). 11 MHz was chosen since it is a suitable time window to capture the ns decay curve. The sync signal of the high repetition rate laser allows for the determination of the macro-times of the luminescence photons during the *T*_off_ period and to construct a decay curve by histogramming these photons. While the *T*_on_ period will contain a combination of fluorescence and luminescence, the decay curve during *T*_off_ can be considered as luminescence only (if the photons in the nanosecond range after switching off the last pulse are ignored). Knowing both the micro- and macro-times, it is possible to gate the photons to extract specific temporal evolutions (see Fig. S2† for a representation of all gating schemes used). For instance, the temporal evolution of the luminescence during *T*_on_ and *T*_off_ in the macro-time domain can be constructed by only using the photons corresponding to the background level in Fig. 1c. A similar temporal evolution of the fluorescence can be constructed in the macro-time domain, allowing to investigate if the equilibrium times for the fluorescence and luminescence are alike. The latter was previously demonstrated by Petty *et al.* and must be the case when all states are connected in a classic three-level system.^12^

To verify whether the microsecond lived luminescent state of DNA-Ag_16_NC is capable of OADF, experiments were conducted by co-illuminating with a primary (520 nm) and a secondary (850 nm) excitation source able to regenerate the fluorescent state (Fig. 1b). Owing to their different optical path lengths, there is a temporal delay (*t*_delay_) between the two pulses on the nanosecond timescale (Fig. 1c). The temporal evolution of OADF in the macro-time domain can be reconstructed by continuously illuminating with the secondary excitation source and micro-time gating the OADF-related photons (Fig. S2c†), as is done for all experiments. Note, however, that Fig. 1b shows the general realization of the experiment, where one can control independently the duration (*T*_on2_) and the macro-time delay (*T*_delay_) of the secondary excitation source with regard to the primary.

### DNA-Ag_16_NC

**Fig. 2:**
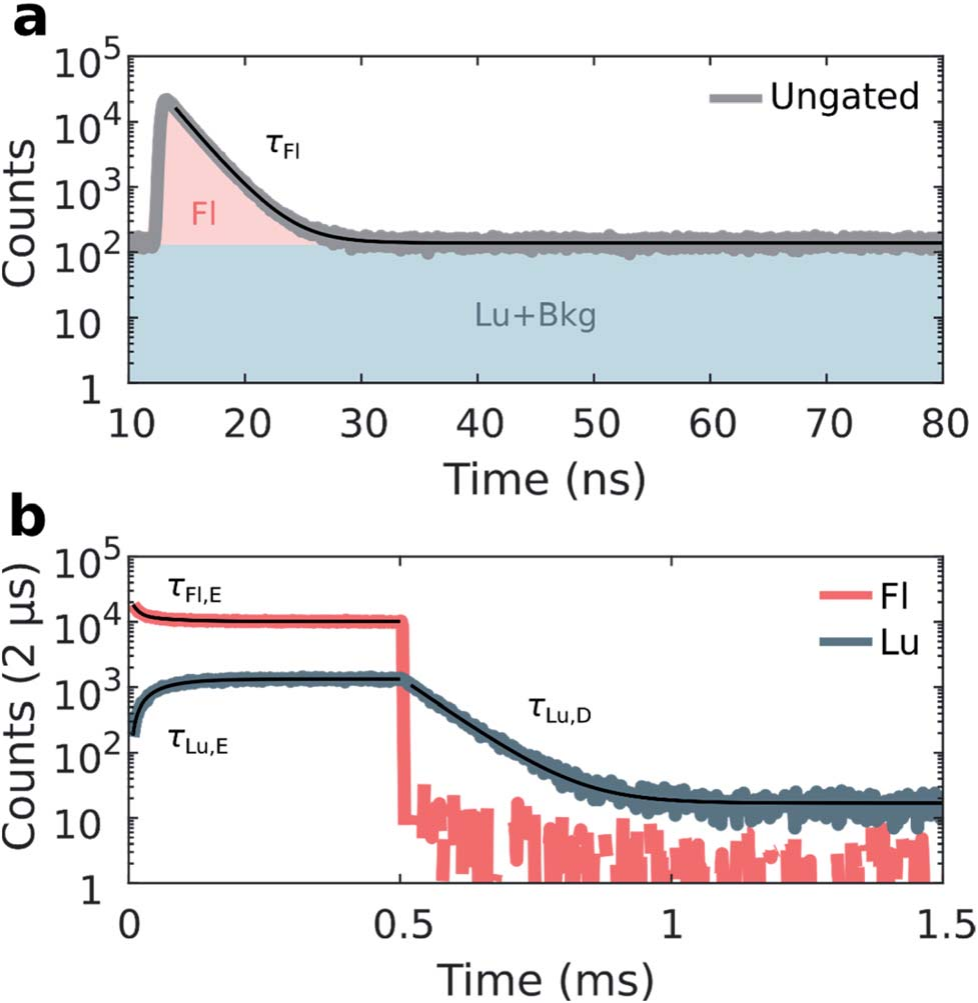
Disentanglement of simultaneously measured fluorescence and luminescence from DNA-Ag_16_NC during 520 nm excitation (1084 W cm^−2^, *f*_Micro_ = 11 MHz, *f*_Macro_ = 500 Hz, *T*_on_ = 0.5 ms, and *T*_off_ = 1.5 ms) in a 10 mM NH_4_OAc D_2_O solution at RT. (a) Ungated micro-times yield the nanosecond fluorescence decay. Coloured areas show micro-time regions used for gating the fluorescence (Fl) and luminescence (Lu) in the macro-time domain. (b) Gated macro-times yield the temporal evolution of the fluorescence and luminescence in the macro-time domain (bin time 2 μs). Fits are shown as black curves.

Details on the synthesis and HPLC purification of the collected DNA-Ag_16_NC fraction can be found in the ESI and Fig. S3.† As reported previously,^13^ DNA-Ag_16_NC has no significant long-lived emission in H_2_O at RT, but is clearly dual emissive in D_2_O with a nano- and microsecond emission component (Fig. S4†).^13^ While the long lived emission bears the hallmarks of phosphorescence, we currently do not feel confident enough to assign it to a spin-forbidden transition. The individual emissive components are spectrally resolved when frozen at 77 K with maxima at around 690 nm and 840 nm, but become hard to separate at RT as the combined emission appears as a single broad feature with a maximum around 750 nm. However, through time-resolved measurements, it is possible to disentangle the spectrally overlapping nanosecond emission from the microsecond emission at RT.^13^ To maximize the luminescence-to-fluorescence ratio, we used an emission filter centered around 850 nm (Fig. S4†). A 10 mM NH_4_OAc D_2_O solution of DNA-Ag_16_NC was illuminated with 520 nm (1084 W cm^−2^, *f*_Micro_ = 11 MHz, *f*_Macro_ = 500 Hz, *T*_on_ = 0.5 ms, and *T*_off_ = 1.5 ms), and micro- and macro-times of the collected photons were recorded. By binning the micro-times of all the photons, the nanosecond decay is immediately obtained (Fig. 2a), with a background composed of luminescence, detector dark counts and after pulsing counts.

Binning all the macro-times directly, yields an ungated intensity trace that represents the combination of both fluorescence and luminescence contributions (see also Fig. S5;† note that Fig. S5† is an example at 77 K, while the data in Fig. 2 is recorded at RT). Since each photon has a micro- and macro-time assigned, it is possible to gate photons based on whether they are in or out of the fluorescence window (approximately from 12 to 35 ns, see Fig. 2a). Furthermore, the fraction of luminescence in the fluorescence window can be calculated (see ESI and Fig. S6† for further details on the disentanglement of both signals). Thus, the macro-time evolution of fluorescence and luminescence can be determined and represented separately (Fig. 2b). Fig. 2b shows that the fluorescence intensity drops with time until the steady-state equilibrium is reached. Similarly, the luminescence intensity increases until it also reaches a steady-state equilibrium (see Fig. S7† for the IRF profile of the burst period). After the last 520 nm laser pulse (at 0.5 ms), a steep drop can be observed, as would be expected for the ns-lived fluorescence. The decay of the luminescence is slower, spanning from tens to hundreds of microseconds at RT and 77 K, respectively. The constant intensity level from *ca.* 1 to 1.5 ms in the luminescence trace (Fig. 2b) is due to detector dark counts. This contribution could be subtracted, but it was neglected here given the low number of counts.

With the fluorescence and luminescence disentangled, it is possible to extract four parameters from the data in Fig. 2: the decay time of the nanosecond fluorescence (*τ*_Fl_), the equilibrium time of fluorescence (*τ*_Fl,E_), the equilibrium time of luminescence (*τ*_Lu,E_), and the decay time of luminescence (*τ*_Lu,D_). Fitting *τ*_Fl_, *τ*_Fl,E_ and *τ*_Lu,E_ bi-exponentially (intensity averaged decay times are represented, see Fig. S8†), and *τ*_Lu,D_ mono-exponentially yields values of: *τ*_Fl_ = 2.17 ns, *τ*_Fl,E_ = 53.7 μs, *τ*_Lu,E_ = 54.1 μs, and *τ*_Lu,D_ = 75.7 μs for the data in Fig. 2.

### Intensity dependence of the equilibrium times

**Fig. 3:**
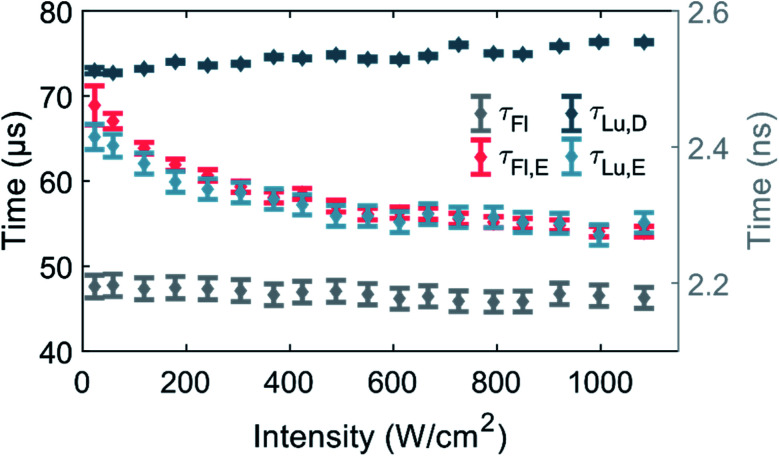
Equilibrium and decay times of DNA-Ag_16_NC a 10 mM NH_4_OAc D_2_O solution at RT as a function of 520 nm excitation intensity. The data point and uncertainty at each excitation intensity represents the weighted average of three measurements. The values of *τ*_Fl,E_, *τ*_Lu,E_ and *τ*_Lu,D_ are associated with the left *Y*-axis, while *τ*_Fl_ is related to the right *Y*-axis. Note that *τ*_Fl_, *τ*_Fl,E_ and *τ*_Lu,E_ are the intensity weighted average values of bi-exponential fits.

Fluorescence and luminescence intensity time traces in the macro-time domain were measured as a function of 520 nm excitation intensity (see Fig. 3 and S9† for exemplary traces) to further our understanding of the photophysics of DNA-Ag_16_NC.

So far, the phenomenological electronic structures of DNA-AgNCs have been described by either a three-^12^ or a four-level model.^19^ The values *τ*_Fl_ and *τ*_Lu,D_ represent post-laser pulse and post-burst processes on the micro- and macro-timescale, respectively, and hence should be excitation intensity-independent. Indeed, in the entire investigated excitation intensity range, *τ*_Fl_ is constant and centered around 2.17 ns. *τ*_Lu,D_ is fairly constant, however, a minor drop from 76 μs to 73 μs can be observed upon going from 1084 W cm^−2^ to 23 W cm^−2^. We believe that this drop is the result of an environmental change, rather than an intensity-dependent phenomenon. During the approximately four hours of measuring, the amount of dissolved molecular oxygen could change, and the concentration of H_2_O would steadily increase by dynamic exchange of D_2_O with atmospheric H_2_O. We have recently demonstrated that an increase in oxygen and H_2_O content lead to a shortening of *τ*_Lu,D_ and this is the most plausible explanation for the change of *τ*_Lu,D_.^13^ Note that the experiment was performed from high to low excitation intensity.

Unlike *τ*_Fl_ and *τ*_Lu,D_, the equilibrium times of the fluorescence (*τ*_Fl,E_) and the luminescence (*τ*_Lu,E_) should be excitation intensity-dependent. For a classic three-level system, *τ*_Fl,E_ would be expected to be equal to *τ*_Lu,E_, as was recently shown for a green-emitting DNA-AgNC, where the excitation intensities were kept below 50 W cm^−2^.^12^*τ*_Fl,E_ and *τ*_Lu,E_ are indeed similar and approach the value of *τ*_Lu,D_ at low excitation intensities.^11^ Shorter equilibrium times are obtained for increasing excitation intensities (Fig. 3). It is worth noticing that both equilibrium times could have been satisfactorily fitted with a mono-exponential function at low excitation intensities, given the limited number of counts. However, at higher excitation intensities, especially above 400 W cm^−2^, *τ*_Fl,E_ was significantly better fitted with a bi-exponential model (see Fig. S8†), thus we chose to fit also *τ*_Lu,E_ bi-exponentially. It is unclear why the equilibrium time *τ*_Fl,E_ is bi-exponential, but a possible explanation could be that there are subsets of populations with different sets of photophysical rates. This suggestion is not unreasonable, since we previously demonstrated for a red-emitting DNA-AgNC that a small fraction of the population had a significantly higher dark state formation yield in poly vinyl alcohol (PVA).^26^ However, this explanation should be considered suggestive at this point and further studies are needed to unravel the origin of the bi-exponential nature of *τ*_Fl,E_, but this is beyond the scope of this paper.

### Simultaneous detection of optically activated delayed fluorescence

Previous demonstrations of OADF have been able to infer the microsecond decay time of the dark state by continuously co-illuminating with a secondary CW laser and extrapolating to zero secondary excitation intensity.^18,19^ In our method, we use the nanosecond response in the micro-time domain to create an OADF gate for constructing the temporal evolution in the macro-time domain, allowing us to directly extract three OADF-related quantities, *τ*_OADF_, *τ*_OADF,E_ and *τ*_OADF,D_, from a single measurement (see below). For OADF measurements on DNA-Ag_16_NC (see Fig. S1 and S7†), a secondary delayed 850 nm pulsed laser (*t*_delay_ = 45 ns, *T*_delay_ = 0 ms, *T*_on_ = 2 ms) is co-illuminating with the primary 520 nm laser (*T*_on_ = 0.5 ms). To block the second excitation wavelength, the detection range was shifted to 640 nm where the luminescence-to-fluorescence ratio is lower (Fig. S4†).

**Fig. 4:**
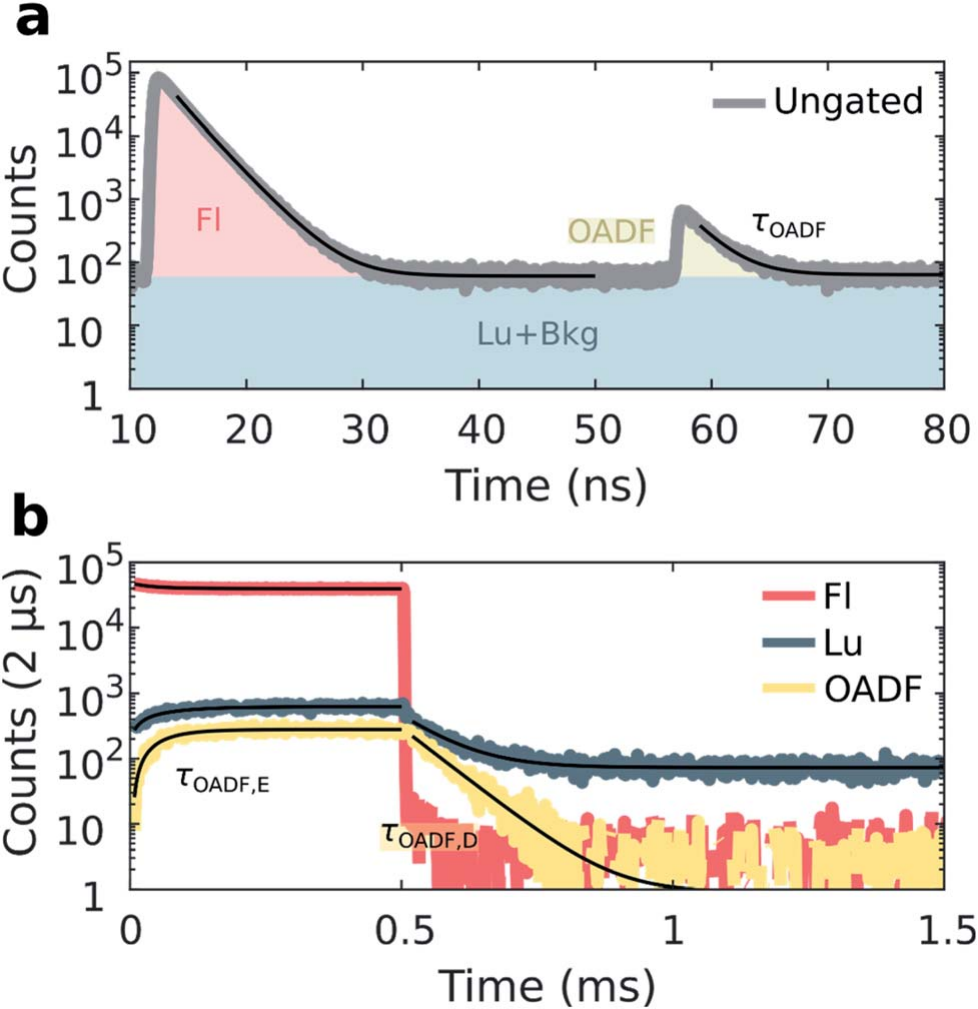
Disentanglement of simultaneously measured fluorescence, luminescence and OADF from DNA-Ag_16_NC during 520 nm (81 W cm^−2^, *f*_Micro_ = 11 MHz, *f*_Macro_ = 500 Hz, *T*_on_ = 0.5 ms, and *T*_off_ = 1.5 ms) and 850 nm (6.3 kW cm^−2^, *f*_Micro_ = 11 MHz, *f*_Macro_ = 500 Hz, and *T*_on_ = 2 ms) co-illumination in a 10 mM NH_4_OAc D_2_O solution at RT. (a) Ungated micro-times yield the primary and secondary fluorescence responses to 520 nm and delayed 850 nm excitation, respectively. An additional micro-time gate, shown in yellow, is used to construct the OADF macro-time domain trace. (b) Micro-time gated macro-times yield fluorescence, luminescence, and OADF temporal evolution traces. Fits are shown as black traces.

The luminescence of DNA-Ag_16_NC was collected during co-illumination in D_2_O at RT, and the ungated micro-times of all photons were binned as shown in Fig. 4a. A fluorescence response is observed when excited by the primary 520 nm laser at 12 ns, and a second smaller response arises at 57 ns due to the secondary 850 nm laser. Similar as for the disentanglement of fluorescence from luminescence, it is possible to separate the additional OADF contribution by micro-time gating (Fig. S2c†). Thus, the primary fluorescence, OADF, and luminescence signals are individually resolved in the macro-time domain (Fig. 4b).

From these measurements, three additional parameters can be extracted: the nanosecond decay of the OADF signal (*τ*_OADF_), the equilibrium time of the OADF state (*τ*_OADF,E_), and the microsecond decay of the OADF state (*τ*_OADF,D_). Fitting every OADF contribution and *τ*_Lu,D_ mono-exponentially and the rest bi-exponentially yields values of: *τ*_Fl_ = 2.17 ns, *τ*_OADF_ = 2.20 ns, *τ*_Fl,E_ = 66.2 μs, *τ*_Lu,E_ = 66.0 μs, *τ*_Lu,D_ = 74.8 μs, *τ*_OADF,E_ = 49.8 μs, and *τ*_OADF,D_ = 65.8 μs for the data in Fig. 4. In agreement with a previous report,^19^ roughly the same nanosecond decay time is observed under primary (*τ*_Fl_) and secondary (*τ*_OADF_) excitation, alluding to the repopulation of the fluorescent state upon 850 nm excitation. The equilibrium time of the state responsible for OADF (*τ*_OADF,E_ = 49.8 μs) is similar but slightly lower than the equilibrium time of the luminescent state (*τ*_Lu,E_ = 66.0 μs), while the macro-domain decay times under additional 850 nm excitation are also similar (*τ*_OADF,D_ = 65.8 μs, *τ*_Lu,D_ = 74.8 μs). Note that a previous report showed that *τ*_OADF,D_ can be multi-exponential, however, the amount of OADF counts in our case is rather low to justify going beyond a mono-exponential fit.^19^ The slight differences between the OADF and luminescence equilibrium and post-burst decay times could be due to the limited numbers of counts for the OADF signal. Despite these minor discrepancies, we feel confident to conclude that the state responsible for OADF is the luminescent state.

Embedment of DNA-AgNCs in a PVA film has previously shown to enhance the overall OADF contribution (area under OADF decay divided by area under primary fluorescence decay like in Fig. 4a).^19^ Also for DNA-Ag_16_NC, the OADF contribution increases from 0.7% in a 10 mM NH_4_OAc D_2_O solution to 2.4% in PVA (Fig. S10†). Furthermore, the ns fluorescence decay lengthens to an intensity-averaged decay time, *τ*_Fl_, of 3.73 ns; *τ*_OADF,D_ also increases to 107 μs. While other DNA-AgNCs have shown upconversion fluorescence (UCF) during sole secondary illumination when embedded in PVA,^18^ DNA-Ag_16_NCs within PVA exhibit negligible UCF at 6.3 kW cm^−2^ (Fig. S10†).

Interestingly, while our results show a seemingly 0.7% OADF efficiency for DNA-Ag_16_NCs in a 10 mM NH_4_OAc D_2_O solution (Fig. 4a), it should be noted that this contains both steady-state and non-steady state contributions (Fig. 4b), and that during *T*_on_ a significant population of luminescent states is built up. One should also realize that the 0.7% is not a single 850 nm pulse efficiency as the OADF-responsible state is subjected to thousands of 850 nm pulses during its lifetime. In fact the value of *τ*_Lu,D_ with the secondary light on (74.8 μs) is basically identical to the value of *τ*_Lu,D_ reported in Fig. 3 (ranging from 76 to 73 μs), where no secondary light was present. These findings are in line with previous work showing that significantly higher secondary excitation intensities are needed to really observe a shortening of *τ*_OADF,D_ (preferably kW cm^−2^ to MW cm^−2^).^18,19^ Another reason for the absence of any significant shortening of *τ*_OADF,D_ is that we did not investigate the wavelength dependency of the OADF process for DNA-Ag_16_NCs and perhaps 850 nm is not the most ideal wavelength.^16^

## Conclusions

We have demonstrated a method for simultaneously measuring nano- and microsecond decay times of a dual emissive DNA-AgNC using TCSPC where each photon is assigned a micro- and a macro-time. Utilizing the combination of micro- and macro-times, we were able to disentangle the spectrally overlapping fluorescence and luminescence of DNA-Ag_16_NC into individual time-resolved contributions. Additionally, the method allows one to determine the equilibrium times for establishing steady-state conditions. Co-illumination measurements with a secondary NIR laser enabled the characterization of the decay and equilibrium times of the state responsible for OADF. The macro-domain decay time of the OADF-related state is similar to the luminescent state decay time, demonstrating for the first time OADF from a luminescent state. In addition to being valuable for characterizing dual emissive DNA-AgNCs in one measurement, we believe that this approach could be of interest for studying a myriad of other systems with emission spanning the nanosecond to millisecond timescale, or where subsequent time-resolved measurements of short- and long-lived emission may alter the true picture, only obtainable from simultaneously measuring both quantities. For this method, the only essential parts are the standard TCSPC detection hardware and a pulsed MHz laser that can operate in a burst mode fashion, eventually complemented with a secondary laser for investigating OADF.

## Data availability

Experimental data and details on the experimental procedures are provided in the ESI.†

## Author contributions

M. B. L. performed all optical measurements and wrote the code for analysing the data. C. C. synthesized and purified the DNA-Ag_16_NC. S. K., M. B. L. and T. V. conceived the experiments. The paper was written with input from M. B. L., S. K., C. C., and T. V.

## Conflicts of interest

There are no conflicts to declare.

## Supplementary Material

SC-013-D2SC01137A-s001
