# Supplementary material for: Probing emission of a DNA-stabilized silver nanocluster from the sub-nanosecond to millisecond timescale in a single measurement
Source: Chem Sci. 2022 Apr 21;13(19):5582–7. doi: 10.1039/d2sc01137a (PMC9116328; doi:10.1039/d2sc01137a)
Supplement: SC-013-D2SC01137A-s001 [file SC-013-D2SC01137A-s001.pdf]

Electronic Supplementary Material (ESI) for Chemical Science.
This journal is © The Royal Society of Chemistry 2022

Supporting Information

Probing Emission of a DNA-Stabilized Silver Nanocluster from the Sub-Nanosecond to Millisecond Timescale in a Single Measurement

Mikkel Baldtzer Liisberg,[a] Stefan Krause,[a] Cecilia Cerretani,[a] and Tom Vosch[a,*]

[a] Nanoscience Center and Department of Chemistry, University of Copenhagen, Universitetsparken 5, 2100 Copenhagen, Denmark, Email: tom@chem.ku.dk

1 Materials and Methods.

1.1 Synthesis of DNA-Ag_{16}NC.

DNA was purchased from Integrated DNA Technologies, D_2O (99.90%) was purchased from Eurisotop, while $AgNO_3$ (≥ 99.998%), ammonium acetate (NH_4OAc, ≥ 98%) and $NaBH_4$ (≥ 99.99%) were acquired from Sigma Aldrich.

DNA-Ag_{16}NCs were synthesized according to the protocol described in Cerretani et al.[1] The freeze-dried DNA was dissolved in D_2O and both $AgNO_3$ and $NaBH_4$ stock solutions were prepared in D_2O. The synthesis was carried out in a 10 mM ammonium acetate (NH_4OAc) D_2O solution (pH 7) by mixing the DNA oligonucleotides with the $AgNO_3$ solution. The mixture was then vortexed and, after 15 minutes, a freshly prepared $NaBH_4$ solution was added. The final ratio between the components was [DNA]: [$AgNO_3$]: [$NaBH_4$] = 25 µM: 187.5 µM: 93.75 µM. After synthesis, the solution was stored in the fridge for 3 days prior to HPLC purification.

The HPLC purification was performed using a preparative HPLC system from Agilent Technologies with an Agilent Technologies 1260 Infinity fluorescence detector, an Agilent Technologies 1100 Series UV-Vis detector, and a Kinetex C18 column (5 µm, 100 Å, 250 × 4.6 mm), equipped with a fraction collector. The mobile phase was a gradient mixture of 35 mM triethylammonium acetate (TEAA) buffer in MilliQ water (A) and methanol (B). The flow rate was 1 mL/min. The elution gradient ranged from 20% to 95% B in 24 min. In the first 2 min, it was kept constant to 20% B, then it was linearly increased to 40% B in 20 min, and finally in the 22-24 min interval, the gradient was rapidly varied from 40% to 95% B. The collection was based on the absorbance at 530 nm. The run was followed by 6 min of washing with 95% B to remove any remaining sample from the column. As shown in the chromatograms in Figure S3, the purified fraction eluted around 16.5-18 min (≈34.5-36% B).

In the end, the collected sample was upconcentrated and solvent-exchanged to 10 mM NH_4OAc D_2O solution by spin-filtration (3 kDa cut-off membrane).

1.2 Confocal Microscope Setup.

A home-built confocal microscope setup was used for all emission measurements and is depicted in Fig. S1. Two slightly different setups were used for single (Fig. S1a) and co-illumination (Fig. S1b) measurements.

1.2.1 Single Wavelength Excitation Setup.

For single wavelength measurements, a fiber coupled (FD7-PM, NKT Photonics) pulsed ~11 MHz continuum white-light laser (SuperK EXTREME EXB-6, NKT Photonics) was used as an excitation source delivering a wavelength of 520 nm by sending the continuum output through an acousto-optic tunable filter (AOTF; SuperK SELECT, NKT Photonics). The output of the fiber was expanded and collimated by a lens system and cleaned up by a 520 nm band-pass filter (FF01-520/5-25, Semrock) and 561 nm short-pass filter (SP01-561RU-25, Semrock) before it was reflected by a 30:70 beam splitter (XF122, Omega Optical) and sent through an oil immersion objective (UPlanSApo 100x, NA = 1.4, Olympus). The objective focused the laser onto the sample, which was positioned on a piezo-driven stage (E-710.4CL, Physik Instrumente); the emission was collected by the same objective. For low temperature (77 K) measurements, an air objective (CPlanFLN 10x, NA = 0.3, Olympus) and temperature-controlled stage (LTS 350 and LNP, Linkam) were used instead. In both cases, the laser light was blocked by a 561 nm long-pass filter (BLP01-561R-25, Semrock) and out-of-focus light was blocked by a 100 μm pinhole. To increase the luminescence contribution relative to the fluorescence, an 850 nm band-pass filter (FF01-850/10-25, Semrock) was inserted in the emission path. The resulting emission was detected on an avalanche photodiode (CD3226, PerkinElmer) connected to a single photon counting board (SPC-830, Becker & Hickl).

1.2.2 Co-Illumination Setup.

For co-illumination measurements, a second path of 850 nm was introduced. The AOTF has two crystals, one for selecting visible wavelengths and another for near infrared (NIR) wavelengths. Thus, the 850 nm output from the NIR port of the AOTF was cleaned with an 850 nm band-pass filter (FF01-850/10-25, Semrock) and directed towards the primary excitation beam. The two beams (520 nm and 850 nm) were combined with a dichroic mirror (TLP01-501-25x36, Semrock) and subsequently reflected by a second dichroic mirror (TLP01-628-25x36, Semrock) and sent through an oil immersion objective (UPlanSApo 100x, NA = 1.4, Olympus). Compared to the single wavelength excitation setup, the 850 nm band-pass filter in the emission path was replaced with a 750 nm short-pass filter. Due to the optical path length differences between the two beams, the secondary pulse appears 45 ns delayed with respect to the primary (Fig. S7).

Since the OADF process is not very efficient, moderate power intensities (kW/cm^2 regime) of the secondary laser were needed. This is the reason for the use of a dichroic mirror instead of the 30:70 beam splitter. In this setup, emission mainly below 700 nm is collected instead of around 850 nm during single wavelength excitation (Fig. S4). The power of the secondary beam was measured directly on top of the microscope with a power meter (S120VC, Thorlabs). The power of the primary beam was controlled with a variable neutral density filter (Thorlabs) and was measured from a reflection in the beam path with the power meter. After measuring the entire range of powers (Fig. 3), the power value at the reflection was noted and subsequently measured on top of the microscope; this factor (i.e., the power value on top of the microscope compared to that at the reflection) was used to calculate every power value on top of the microscope.

1.2.3 Generation of Burst Profiles.

The temporal profiles of the light sources are controlled by driving the AOTF crystals with variable external signals. Each AOTF crystal is connected to an external radio frequency (RF) driver; these RF drivers are in turn connected to separate modules (SuperK COMMAND, NKT Photonics) that are able to drive and synchronize fast wavelength switching or amplitude modulation of the output of the AOTF based on input from an external source. As an external source, we used a delay generator (DG; DG535, Stanford Research Systems). The DG has a variable internal trigger that defines the repetition rate of each burst (f_{Macro}). Two ports of the DG, which outputs TTL pulses with variable width and delay, are connected to each of the SuperK COMMAND modules. Thus, variable on (T_{on}) and off (T_{off}) times, as well as variable delays (T_{delay}), can be obtained for both the primary and secondary excitation paths independently. It was found that the SuperK COMMAND modules worked best using the high impedance output of the DG in order to obtain the right temporal profiles. In order to be able to correctly overlay all the bursts during the analysis, a second DG is introduced, due to the limited number of TTL output ports on the DG535. The second DG is triggered at the beginning of each burst and sends a 50 ns TTL pulse to the multichannel router of the TCSPC board, which is used to overlay all the 2 ms cycles.

2 Data Analysis.

Fitting of decays was performed in Origin 2020, while all other data analysis was conducted in MATLAB R2020b. In all bi-exponential fits, where more than one decay curve was present (e.g., in the excitation intensity series of Fig. 3) the decay times were globally linked. Only for $\tau_{\mathrm{Lu,E}}$, an

additional upper boundary constraint of 0 was set for the amplitudes of the bi-exponential fits (Fig. 3).

2.1.1 Overlapping of Macro-Times.

In regular TCPSC experiments, nanosecond decays are directly obtained by generating histograms of the micro-times. In this burst-mode approach, many individual bursts are recorded. There is an initial build-up of long-lived emission during the on period of the excitation source, and a subsequent decay when the excitation source is turned off (Fig. S5b). By precisely knowing the beginning of each burst, it is possible to overlap all of the individual bursts (Fig. S5c), which is done with a self-written MATLAB algorithm (Section 4.1). This allows us to determine the nanosecond decays (Fig. S5a) by utilizing the micro-times and the high repetition rate sync of the excitation source, as well as microsecond equilibrium and decay times using the macro-times with the timing information from the second DG.

2.1.2 Gating of Micro- and Macro-Times.

To gate the photons in the macro-time domain, micro-time information can be used (Fig. S2 and Section 4.2). Considering the fluorescence micro-gate (Fig. 2a) as an example, a fluorescence window is defined encompassing all fluorescence from which a logical mask is generated (from 10 to 30 ns); the opposite logical mask will in turn gate luminescence photons (Fig. 6a). Directly binning the macro-times of these photons will yield a trace on the microsecond timescale predominately composed of fluorescence photons. However, within this gate, some luminescence photons are also included. To correct for this, the luminescence gate is used to prepare the luminescence macro-time trace, which is entirely free of fluorescence (Fig. 6b). The ratio between the widths of fluorescence and luminescence micro-gates is calculated and represents the fraction of luminescence within the fluorescence micro-gate (Fig. S6c, d). Thus, the fluorescence macro-time trace can be corrected by subtracting this ratio multiplied by the luminescence macro-time trace. A similar correction is done for the luminescence macro-time trace, but by adding the above-mentioned ratio (Fig. S6e, f).

3 Additional Figures

3.1 Optical Setup

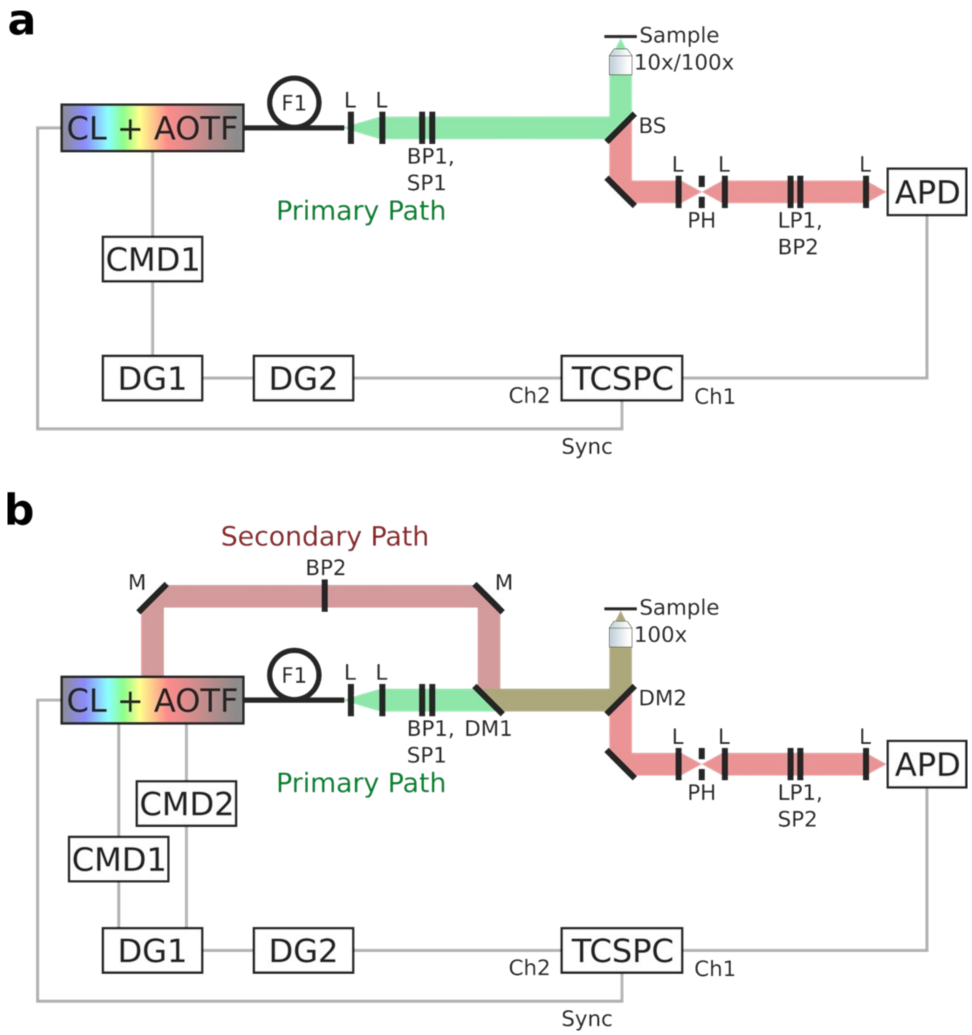

Fig. S1: Overview of the setups used for simultaneously measuring fluorescence and luminescence during (a) single excitation and (b) co-illumination. CL (SuperK EXTREME EXB-6, NKT Photonics), AOTF (SuperK SELECT, NKT Photonics), F1 (FD7-PM, NKT Photonics), CMD1/CMD2 (SuperK COMMAND, NKT Photonics), DG1/DG2 (DG535, Stanford Research Systems), TCPSC (SPC-830, Becker & Hickl), BP1 (FF01-520/5-25, Semrock), BP2 (FF01-850/10-25, Semrock), SP1 (SP01-561RU-25, Semrock), SP2 (750 nm SP), LP1 (BLP01-561R-25, Semrock), DM1(TLP01-501-25x36, Semrock), DM2 (TLP01-628-25x36, Semrock), BS (30:70, XF122, Omega Optical) 10x (CPlanFLN 10x, NA = 0.3, Olympus), 100x (UPlanSApo 100x NA = 1.4, Olympus), APD (CD3226, PerkinElmer), and M (PF10-03-P01, Thorlabs).

3.2 Gating Schemes

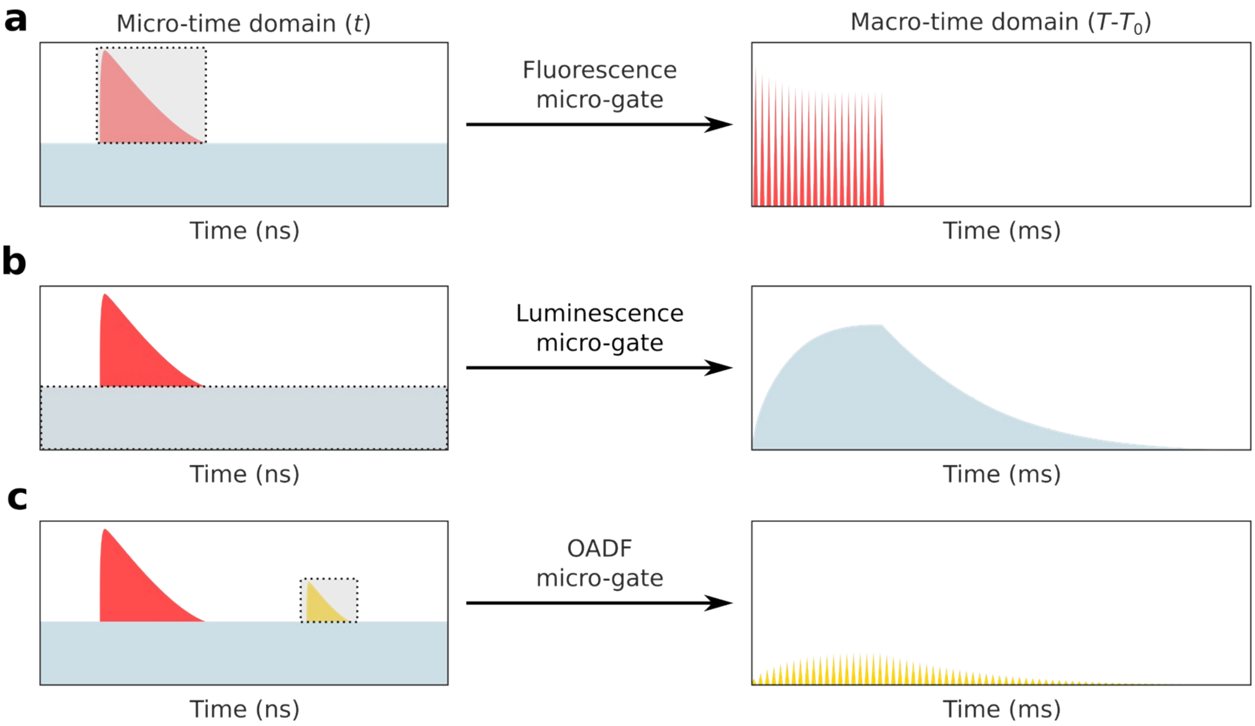

Fig. S2: Gating schemes used for disentangling fluorescence, luminescence, and OADF contributions in the micro- and macro-time domains. Micro-gating the (a) fluorescence, (b) luminescence, or (c) OADF yields disentangled traces in the macro-time domain.

3.3 HPLC Chromatograms

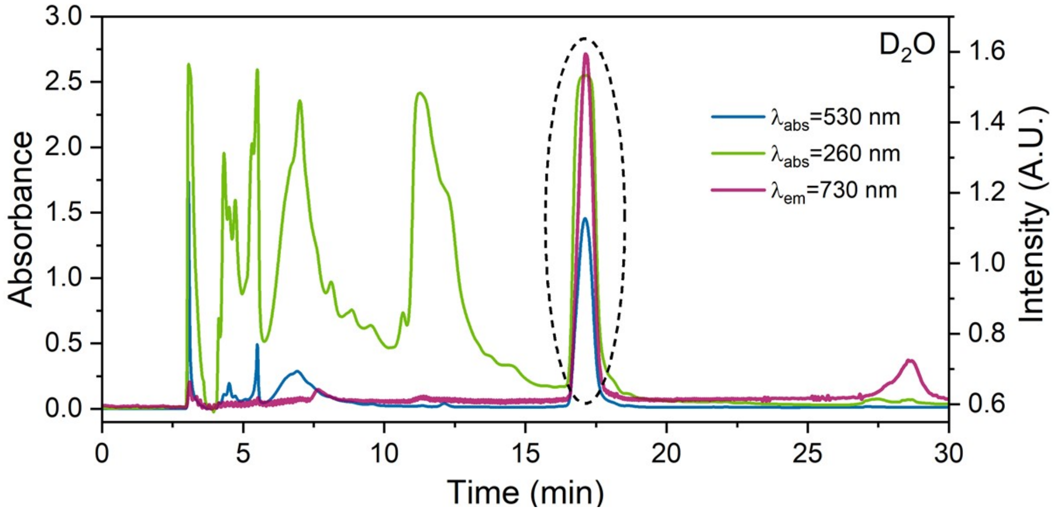

Fig. S3: HPLC chromatograms of DNA-Ag_{16}NC monitoring the main absorption peak of the AgNC at 530 nm (blue), the DNA absorption at 260 nm (light green), and the emission (purple) of

the DNA-Ag_{16}NC at 730 nm (λ_{exc} = 530 nm). The fraction collected between 16.5-18 min (≈34.5-36 % B) is the sample described in the manuscript.

3.4 Emission Spectra

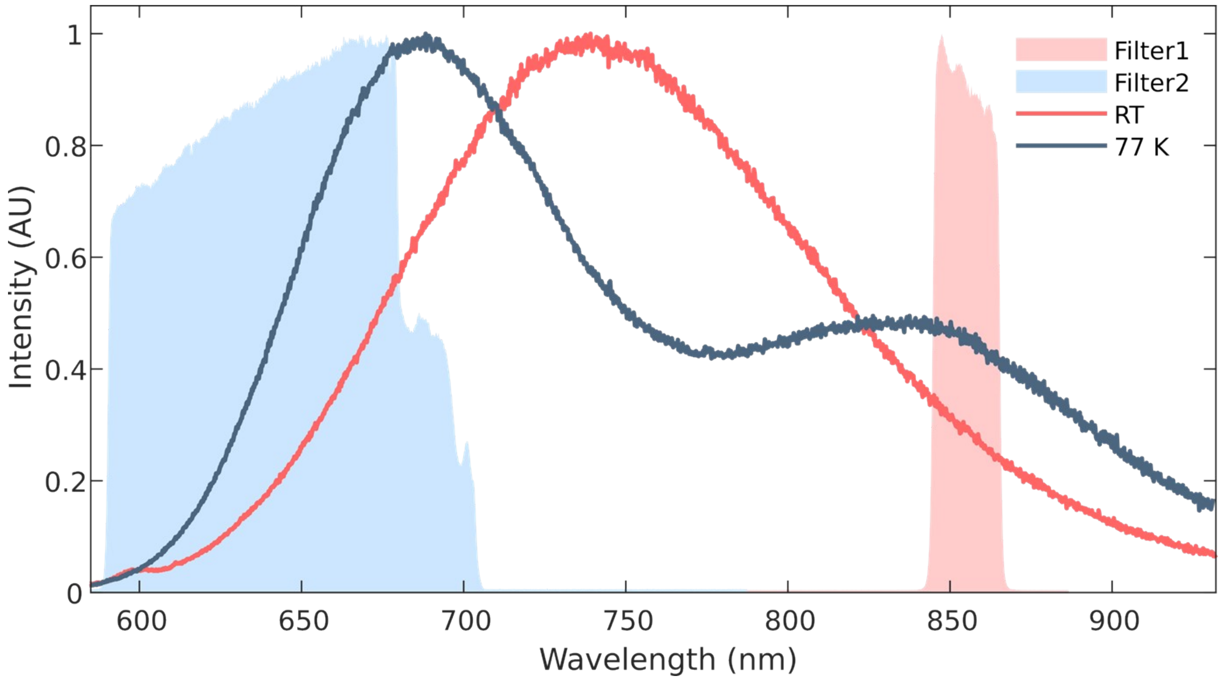

Fig. S4: Emission spectra of DNA-Ag_{16}NC in 10 mM NH_4OAc D_2O at RT and 77 K and spectral ranges of filter settings used for time-resolved measurements. Filter1 was used for measurements during single illumination (Fig. 2), while Filter2 was used for co-illumination measurements (Fig. 4). The spectral profiles of the filters were measured on the home-built microscope setup (Fig. S1), but with light from an incandescent light bulb directed through a spectrograph (SP 2356 spectrometer, 300 grooves/mm, Acton Research) onto a nitrogen cooled CCD camera (SPEC-10:100B/LN-eXcelon, Princeton Instruments), instead of towards the APD. Note that the spectra of the filters were not intensity corrected, thus they do not represent the true transmission profiles of the filters. Emission spectra of DNA-Ag_{16}NC were reproduced from Cerretani et al.[1]

3.5 Overlapping Procedure

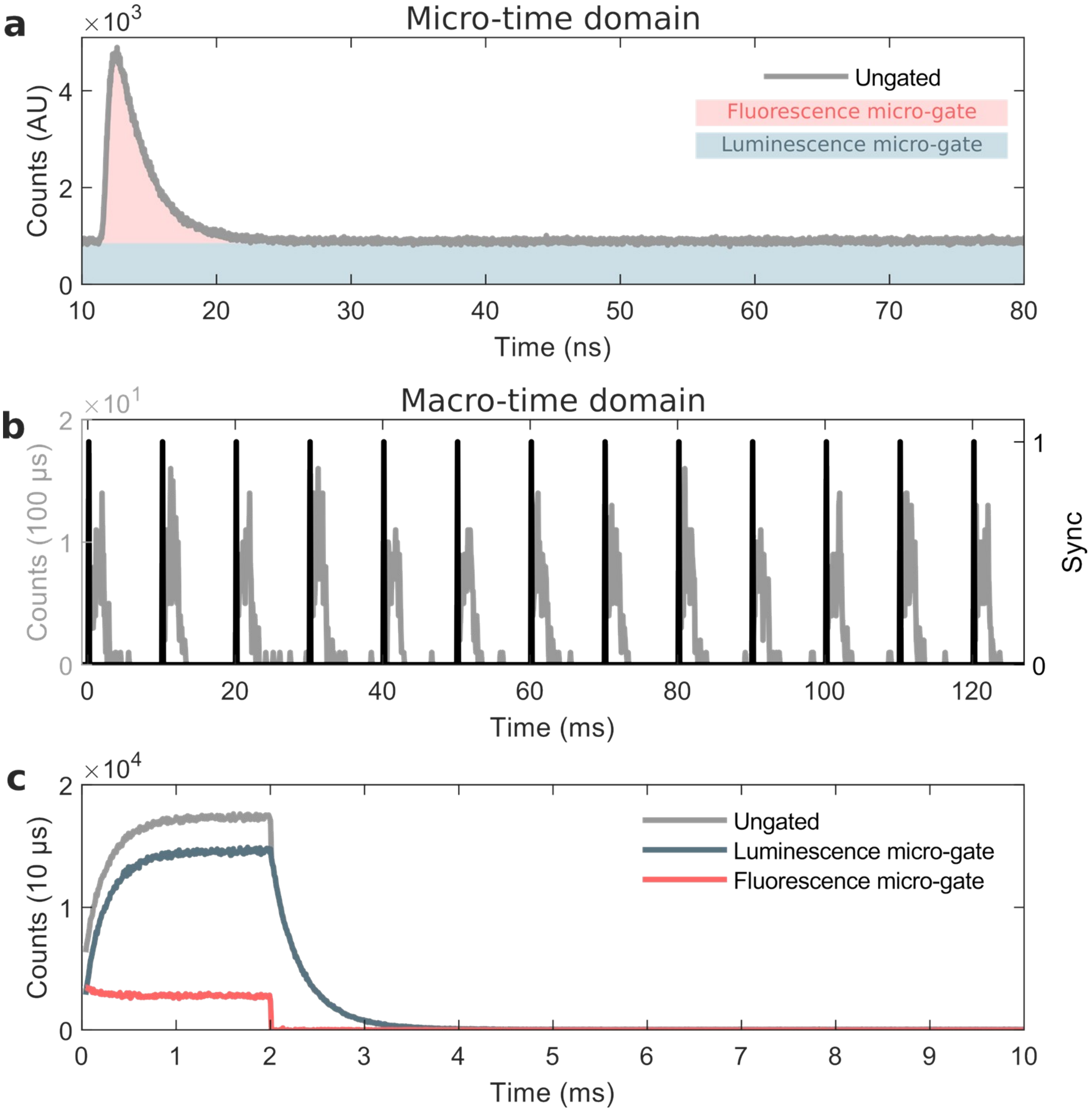

Fig. S5: Overview of the data processing steps to yield short (micro-time domain) and long (macro-time domain) dynamics from a DNA-Ag_{16}NC sample in D_2O at 77 K. The sample was excited at 520 nm (f_{Micro} = 11 MHz, f_{Macro} = 100 Hz, T_{on} = 2 ms, and T_{off} = 8 ms). (a) Micro-time domain showing the nanosecond decay. Gates to probe the fluorescence and luminescence are shown as shaded areas. (b) Macro-time domain, showing a short section of a 200 s trace. Sync pulses are used as accurate measures of the beginning of every burst cycle; between each sync pulse, the

build-up and decay of luminescence can be seen. (c) Using the sync pulses, each individual build-up and decay of the 200 s trace are overlapped. When ungated, the dynamics represent a combination of fluorescence and luminescence, whereas gating the photons according to either the fluorescence or luminescence micro-times disentangles the dynamics.

3.6 Micro-Gating Procedure

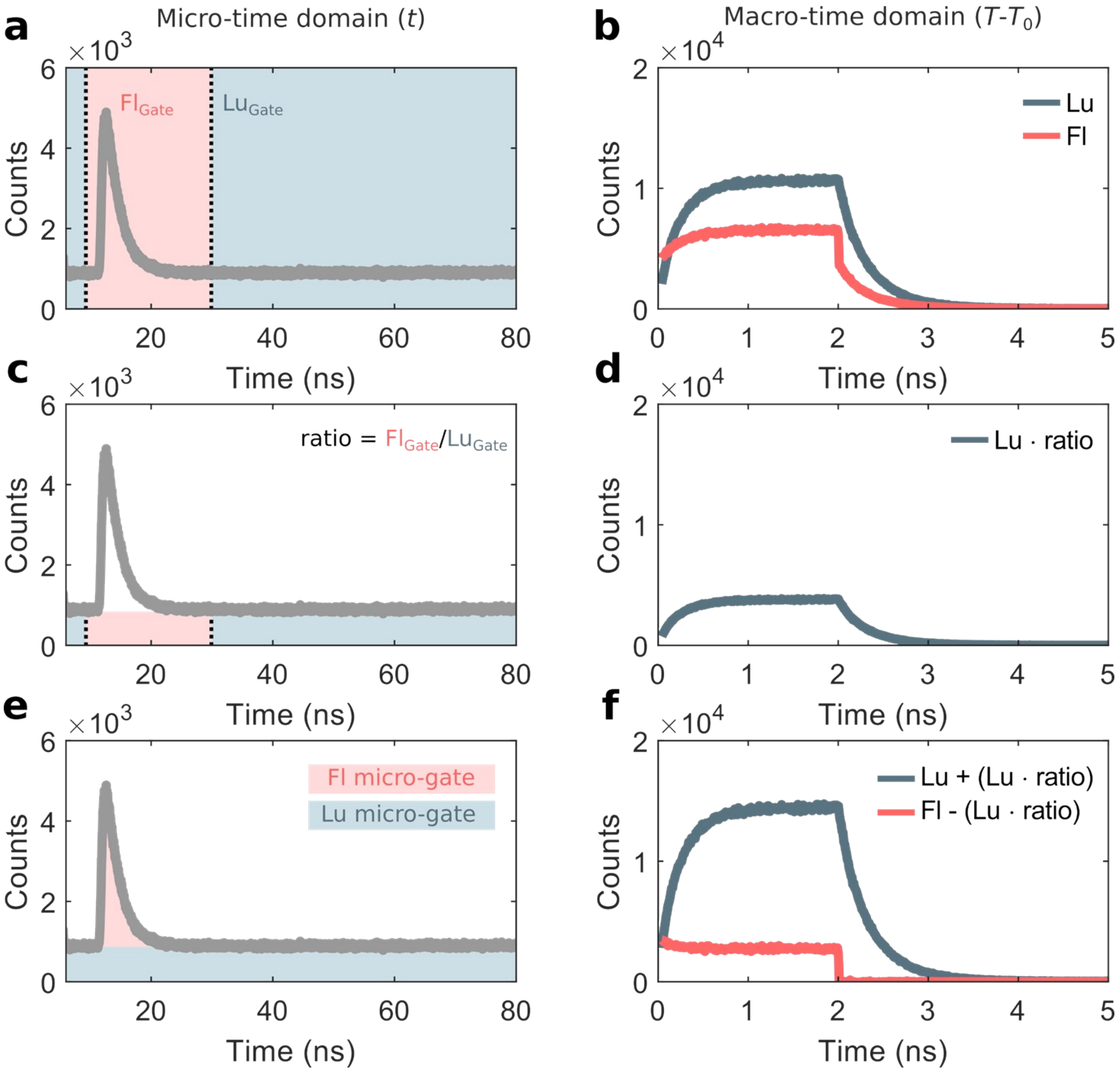

Fig. S6: Overview of the micro-gating procedure used to disentangle macro-time domain fluorescence and luminescence contributions. The exemplary data is the same as in Fig. S5. (a) Fluorescence (Fl_{Gate}) and luminescence (Lu_{Gate}) gates are defined in the micro-time domain. (b) Binning the macro-times according to the gates in (a) yields an entangled fluorescence trace and

an underestimated luminescence trace. (c) The ratio between the width (x-axis) of the fluorescence to luminescence gate is calculated. (d) The ratio, Fl_{Gate}/Lu_{Gate}, multiplied by the luminescence trace yields the amount of luminescence included in Fl_{gate}. (e) The Fl and Lu micro-gates, used throughout the paper, represents the properly disentangled gates. (f) The Fl and Lu micro-gates yields disentangled and corrected fluorescence and luminescence traces in the macro-time domain by subtracting or adding the trace of (d) to the traces of (b), respectively.

3.7 Instrument Response Functions

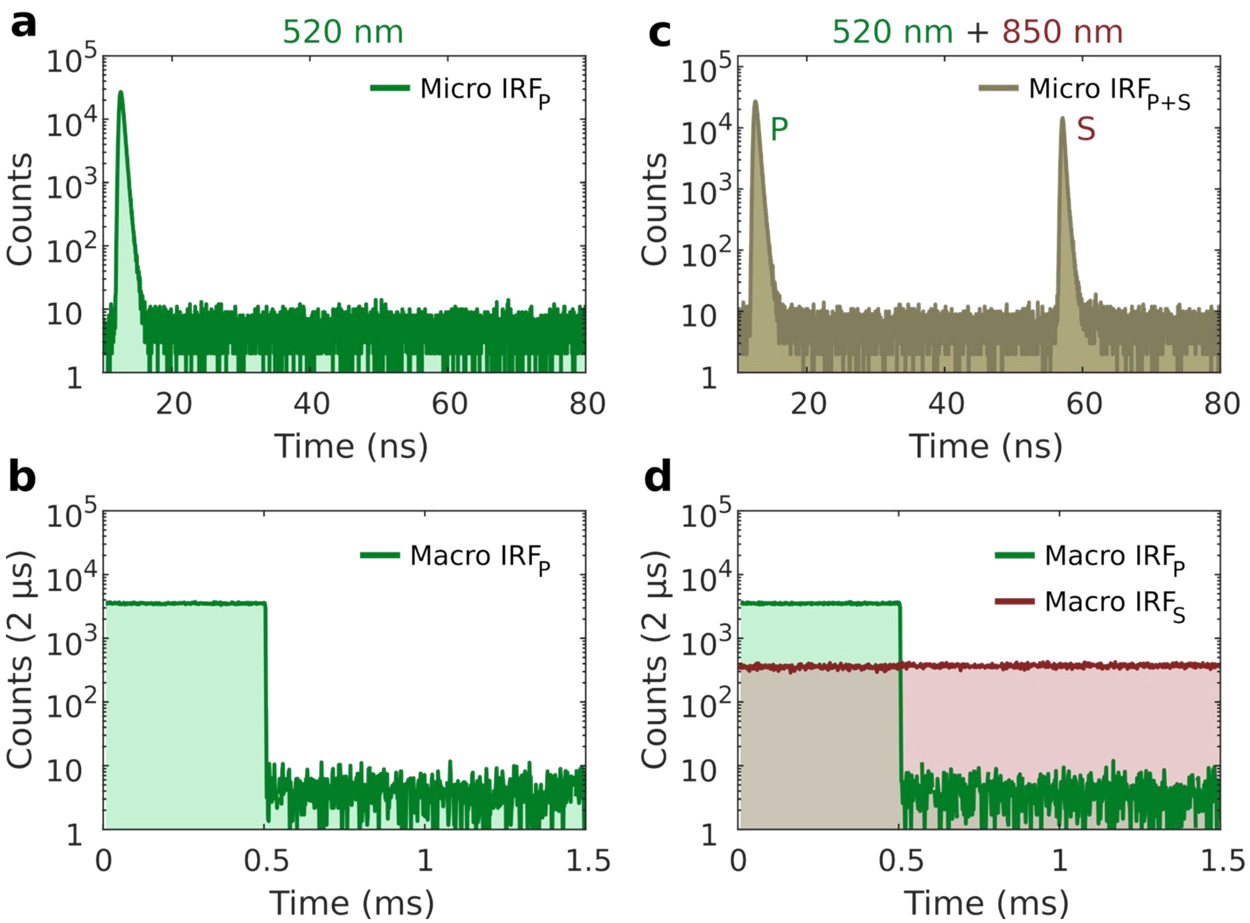

Fig. S7: IRFs of micro- and macro-time domains under (a, b) primary (520 nm) and (c, d) primary + secondary co-illumination (520 nm + 850 nm). The primary 520 nm light source had the following temporal quantities: f_{Micro} = 11 MHz, f_{Macro} = 500 Hz, T_{on} = 0.5 ms, and T_{off} = 1.5 ms. The 850 nm light source had f_{Micro} = 11 MHz, but was otherwise continuously illuminated, as can be seen from (d).

3.8 Mono- vs Bi-Exponential Fits

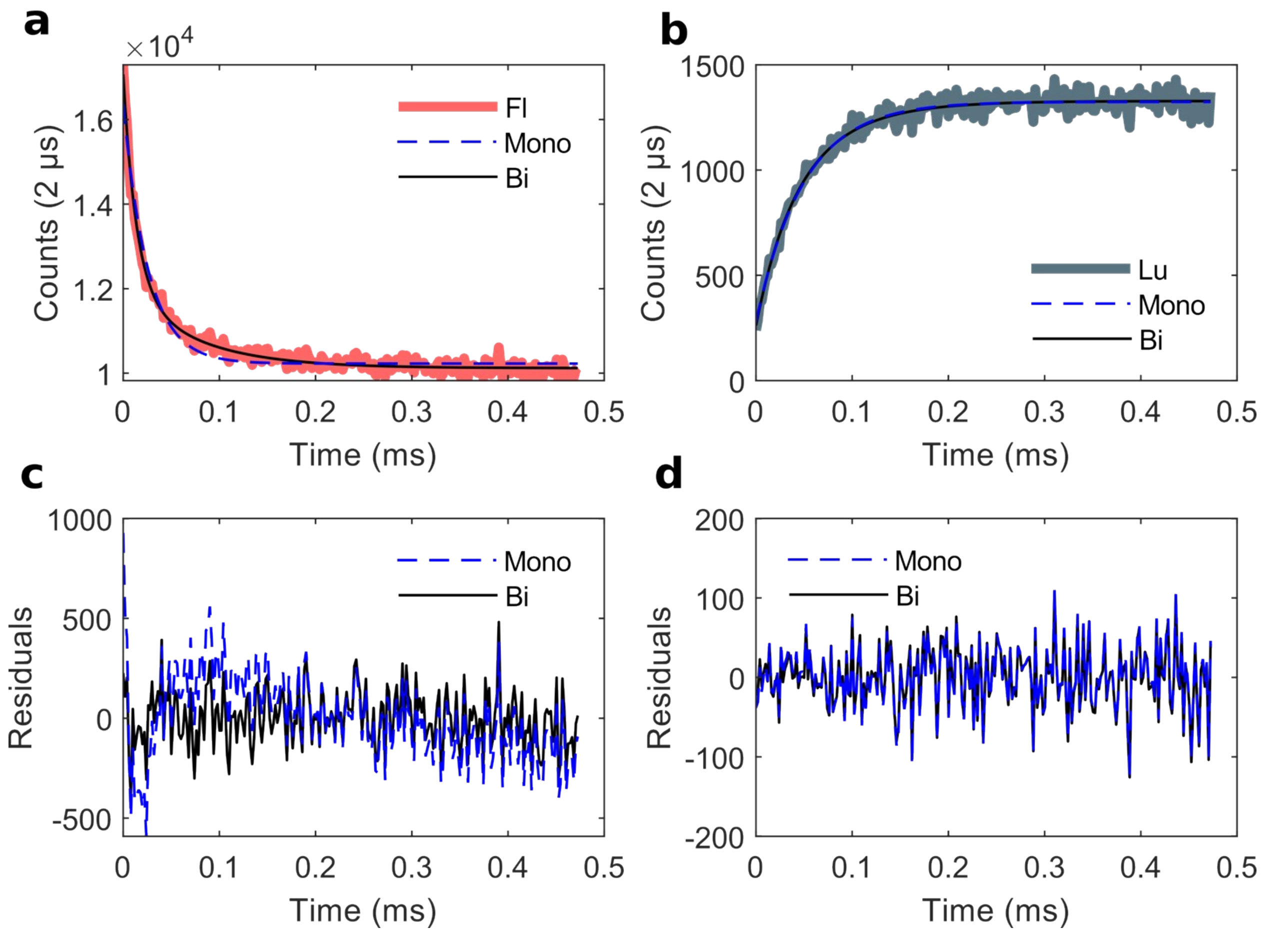

Fig. S8: Data from Fig. 2 showing the effect of fitting the equilibrium times either mono- or bi-exponentially. Equilibration of (a) fluorescence and (b) luminescence on the macro-time scale fitted either with a mono- or bi-exponential function. (c, d) Residuals of the corresponding fits highlight the need for an additional component to adequately fit the fluorescence equilibration, while the luminescence is well described by a single component. For consistency, we decided to represent in Fig. 3 the data where both equilibration times were fitted bi-exponentially.

3.9 Exemplary Luminescence and Fluorescence Macro-Time Traces

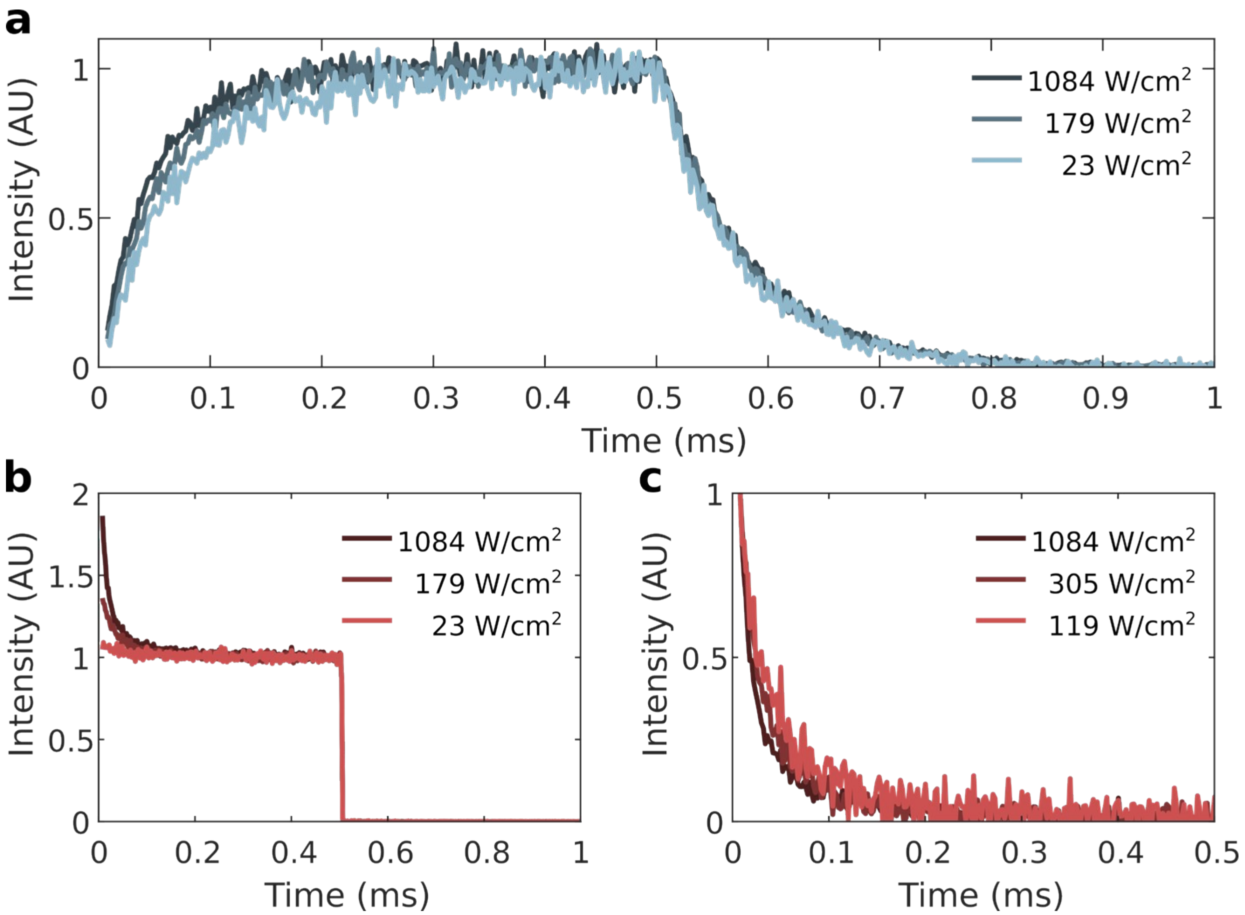

Fig. S9: Exemplary luminescence and fluorescence macro-time traces of DNA-Ag_{16}NC in D_2O at RT during 520 nm excitation (23-1084 W/cm^2, f_{Micro} = 11 MHz, f_{Macro} = 500 Hz, T_{on} = 0.5 ms, and T_{off} = 1.5 ms). (a) Luminescence macro-time traces showing a faster equilibration time at increasing excitation intensities. To make the traces comparable, they were first background subtracted and subsequently normalized to the mean value at the plateau (steady-state). (b) Fluorescence macro-time traces showing faster equilibration times and larger amplitudes for increasing excitation intensities. The traces were normalized to the mean value at the steady-state plateau. (c) To highlight the changes in equilibration times of the fluorescence, the traces were first subtracted by the mean value at the plateau and subsequently normalized to the peak value. Note that the traces shown in (c) are at different excitation intensities, compared to (b), because of the low amplitude seen at the lowest excitation intensities.

3.10 OADF and UCF of DNA-Ag_{16}NC in PVA

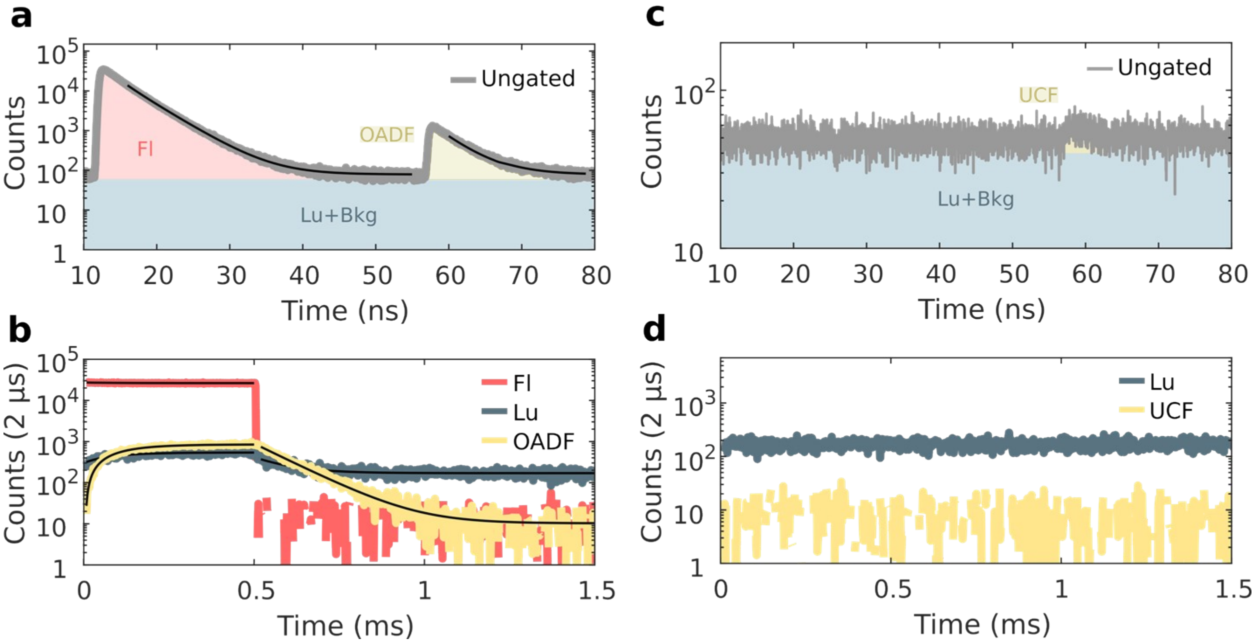

Fig. S10: (a, b) OADF and (c, d) UCF measurements of DNA-Ag_{16}NCs in PVA dissolved in D_2O at RT. For OADF measurements, the sample was co-illuminated with 520 nm (6.6 W/cm^2, f_{Micro} = 11 MHz, f_{Macro} = 500 Hz, T_{on} = 0.5 ms, and T_{off} = 1.5 ms) and 850 nm (6.3 kW/cm^2, f_{Micro} = 11, f_{Macro} = 500 Hz, and T_{on} = 2 ms), while the same sample was only illuminated with 850 nm during UCF measurements. In PVA, as compared to 10 mM NH_4OAc D_2O solution, the ns decay times are longer, leaving a smaller region for micro-gating the luminescent photons, resulting in a higher background level for the luminescent trace in the macro-time domain. The latter could be addressed by changing the repetition rate for example. From the OADF measurements, the ns decays are fitted bi-exponentially (with intensity averaged decays times reported) and the µs equilibrium traces/decays mono-exponentially: τ_{Fl} = 3.73 ns, τ_{OADF} = 3.65 ns, $\tau_{Fl,E}$ = 94.7 µs $\tau_{Lu,E}$ = 98.1 µs, $\tau_{Lu,D}$ = 96.7 µs, $\tau_{OADF,E}$ = 97.3 µs, and $\tau_{OADF,D}$ = 106.8 µs. From the UCF measurements, only a negligible signal is seen in the micro-time domain.

4 MATLAB Code

4.1 Overlapping and Micro-Gating of Trace

```
function [x_Micro, y_Micro, x_Macro, y_Macro_Fl, y_Macro_Lu, y_Macro_OADF] =
Overlap_MicroMacro_Decays(FileName, Mode)
%%%%%
%
%This function takes an asc file and extracts the micro-times to prepare a
%ns trace and the macro-times to prepare a µs-s trace. The asc file is
%composed of three columns: the first represents the macro-times, the
%second the micro-times, and the third the channel nr (0 for photon
%detection and 1 for sync). The function further micro-gates the photons
%according to fluorescence (Fl), luminescence (Lu), or optically activated
%delayed fluorescence (OADF).
%
%Input:
%   FileName: Name of the asc file as a string
%   Mode: Gate either Fl and Lu ('FlLu') or Fl, Lu, and OADF ('FlLuOADF')
%
%%%%%


%%%% Loading of data %%%%
data = importdata(FileName);

Sync = data(:, 3) == 1;
Dec = data(~Sync, 1:2);
Sync_Idx = find(Sync == 1);

%%%% Micro-times analysis %%%%
[y_Micro, ~] = hist(Dec(:, 2), linspace(1, 4096, 4096));
x_Micro = linspace(0, 90, 4096);

%%%% Macro-times analysis %%%%
%%% Overlapping of macro-time decays %%%
n = length(Sync_Idx);
Overlapped = zeros(length(data), 2);

for i = 2 : n - 1

    Overlapped(Sync_Idx(i) + 1 : Sync_Idx(i + 1) - 1, 1) =...
        data(Sync_Idx(i) + 1 : Sync_Idx(i + 1) - 1, 1) - data(Sync_Idx(i));

    Overlapped(Sync_Idx(i) + 1 : Sync_Idx(i + 1) - 1, 2) =...
        data(Sync_Idx(i) + 1 : Sync_Idx(i + 1) - 1, 2);


end

Overlapped = Overlapped(Overlapped(:, 1) ~=0, :);

%%% Gating of macro-times from micro-times %%%
Gate_Start_Fl = 10;
Gate_End_Fl = 35;
if strcmp(Mode, 'FlLu')

    [x_Macro, y_Macro_Fl, y_Macro_Lu] = GateFlLu(Overlapped, Gate_Start_Fl,
Gate_End_Fl);
    y_Macro_OADF = [];

elseif strcmp(Mode, 'FlLuOADF')
```

```
    Gate_Start_OADF = 56;
    Gate_End_OADF = 70;
    [x_Macro, y_Macro_Fl, y_Macro_OADF, y_Macro_Lu] = GateFlLuOADF(Overlapped,
Gate_Start_Fl, Gate_End_Fl, Gate_Start_OADF, Gate_End_OADF);

end

end
```

4.2 Gating of Fluorescence and Luminescence

```
function [x_Macro, y_Macro_Fl, y_Macro_Lu] = GateFlLu(Overlapped, Gate_Start_Fl,
Gate_End_Fl)
%%%%%
%
%This function takes the 'Overlapped' photons from the
%'Overlap_MicroMacro_Decays' function and micro-gates the photons according
%fluorescence or luminescence.
%
%Input:
%   Overlapped: Matrix with overlapped macro- and micro-times.
%   Gate_Start_Fl: Start position of the fluorescence micro window in ns
%   Gate_End_Fl: End position of the fluorescence micro window in ns
%
%%%%%

x_Micro = linspace(0, 90, 4096)';

%%%% Fluorescence gate %%%%
[Gate_Start_Ind_Fl, ~] = find(x_Micro > Gate_Start_Fl);
[Gate_End_Ind_Fl, ~] = find(x_Micro < Gate_End_Fl);

Gate_Fl = logical((Overlapped(:, 2) > Gate_Start_Ind_Fl(1)) .* ...
                  (Overlapped(:, 2) < Gate_End_Ind_Fl(end)));

%%%% Luminescence gate %%%%
Gate_Start_Lu1 = 6;
Gate_Start_Lu2 = Gate_End_Fl;
Gate_End_Lu1 = Gate_Start_Fl;
Gate_End_Lu2 = 82;

[Gate_Start_Ind_Lu1, ~] = find(x_Micro > Gate_Start_Lu1);
[Gate_Start_Ind_Lu2, ~] = find(x_Micro > Gate_Start_Lu2);
[Gate_End_Ind_Lu1, ~] = find(x_Micro < Gate_End_Lu1);
[Gate_End_Ind_Lu2, ~] = find(x_Micro < Gate_End_Lu2);

Gate_Lu1 = logical((Overlapped(:, 2) > Gate_Start_Ind_Lu1(1)) .*...
                   (Overlapped(:, 2) < Gate_End_Ind_Lu1(end)));
Gate_Lu2 = logical((Overlapped(:, 2) > Gate_Start_Ind_Lu2(1)) .* ...
                   (Overlapped(:, 2) < Gate_End_Ind_Lu2(end)));

Gate_Lu = logical(Gate_Lu1 + Gate_Lu2);

%%%% Histogram %%%%
HistRange = linspace(0, 2*10^-3, 1000);
[y_Macro_Fl, x_Macro] = hist(Overlapped(Gate_Fl, 1), HistRange);
[y_Macro_Lu, ~] = hist(Overlapped(Gate_Lu, 1), HistRange);

%%%% Correct for luminescence in the fluorescence trace %%%%
Length_Fl = length(Gate_Start_Ind_Fl(1) : Gate_End_Ind_Fl(end));
Length_Lu = length(Gate_Start_Ind_Lu1(1): Gate_End_Ind_Lu1(end)) +...
            length(Gate_Start_Ind_Lu2(1): Gate_End_Ind_Lu2(end));
```

```
Ratio = Length_Fl / Length_Lu;

y_Macro_Fl = y_Macro_Fl - Ratio * y_Macro_Lu;
y_Macro_Lu = y_Macro_Lu + Ratio * y_Macro_Lu;

end
```

4.3 Gating of Fluorescence, Luminescence and OADF

```
function [x_Macro, y_Macro_Fl, y_Macro_OADF, y_Macro_Lu] = GateFlLuOADF(Overlapped,
Gate_Start_Fl, Gate_End_Fl, Gate_Start_OADF, Gate_End_OADF)
%%%%%
%
%This function takes the 'Overlapped' photons from the
%'Overlap_MicroMacro_Decays' function and micro-gates the photons according
%fluorescence, luminescence, and OADF.
%
%Input:
%   Overlapped: Matrix with overlapped macro- and micro-times.
%   Gate_Start_Fl: Start position of the fluorescence micro window in ns
%   Gate_End_Fl: End position of the fluorescence micro window in ns
%   Gate_Start_OADF: Start position of the OADF micro window in ns
%   Gate_End_OADF: End position of the OADF micro window in ns
%
%%%%%

x_Micro = linspace(0, 90, 4096)';

%%%% Fluorescence gate %%%%
[Gate_Start_Ind_Fl, ~] = find(x_Micro > Gate_Start_Fl);
[Gate_End_Ind_Fl, ~] = find(x_Micro < Gate_End_Fl);

Gate_Fl = logical((Overlapped(:, 2) > Gate_Start_Ind_Fl(1)) .* ...
                  (Overlapped(:, 2) < Gate_End_Ind_Fl(end)));

%%%% OADF gate %%%%
[Gate_Start_Ind_OADF, ~] = find(x_Micro > Gate_Start_OADF);
[Gate_End_Ind_OADF, ~] = find(x_Micro < Gate_End_OADF);

Gate_OADF = logical((Overlapped(:, 2) > Gate_Start_Ind_OADF(1)) .* ...
                    (Overlapped(:, 2) < Gate_End_Ind_OADF(end)));

%%%% Luminescence gate %%%%
Gate_Start_Lu1 = 6;
Gate_Start_Lu2 = Gate_End_Fl;
Gate_Start_Lu3 = Gate_End_OADF;
Gate_End_Lu1 = Gate_Start_Fl;
Gate_End_Lu2 = Gate_Start_OADF;
Gate_End_Lu3 = 82;


[Gate_Start_Ind_Lu1, ~] = find(x_Micro > Gate_Start_Lu1);
[Gate_Start_Ind_Lu2, ~] = find(x_Micro > Gate_Start_Lu2);
[Gate_Start_Ind_Lu3, ~] = find(x_Micro > Gate_Start_Lu3);
[Gate_End_Ind_Lu1, ~] = find(x_Micro < Gate_End_Lu1);
[Gate_End_Ind_Lu2, ~] = find(x_Micro < Gate_End_Lu2);
[Gate_End_Ind_Lu3, ~] = find(x_Micro < Gate_End_Lu3);

Gate_Lu1 = logical((Overlapped(:, 2) > Gate_Start_Ind_Lu1(1)) .*...
                   (Overlapped(:, 2) < Gate_End_Ind_Lu1(end)));
Gate_Lu2 = logical((Overlapped(:, 2) > Gate_Start_Ind_Lu2(1)) .* ...
                   (Overlapped(:, 2) < Gate_End_Ind_Lu2(end)));
Gate_Lu3 = logical((Overlapped(:, 2) > Gate_Start_Ind_Lu3(1)) .* ...
                   (Overlapped(:, 2) < Gate_End_Ind_Lu3(end)));
```

```
Gate_Lu = logical(Gate_Lu1 + Gate_Lu2 + Gate_Lu3);

%%%% Histogram %%%%
HistRange = linspace(0, 2*10^-3, 1000);
[y_Macro_Fl, x_Macro] = hist(Overlapped(Gate_Fl, 1), HistRange);
[y_Macro_OADF, ~] = hist(Overlapped(Gate_OADF, 1), HistRange);
[y_Macro_Lu, ~] = hist(Overlapped(Gate_Lu, 1), HistRange);

%%%% Correct for luminescence in the fluorescence and OADF traces %%%%
Length_Fl = length(Gate_Start_Ind_Fl(1) : Gate_End_Ind_Fl(end));
Length_OADF = length(Gate_Start_Ind_OADF(1) : Gate_End_Ind_OADF(end));
Length_Lu = length(Gate_Start_Ind_Lu1(1): Gate_End_Ind_Lu1(end)) +...
            length(Gate_Start_Ind_Lu2(1): Gate_End_Ind_Lu2(end)) +...
            length(Gate_Start_Ind_Lu3(1): Gate_End_Ind_Lu3(end));

Ratio_Fl = Length_Fl / Length_Lu;
Ratio_OADF = Length_OADF / Length_Lu;

y_Macro_Fl = y_Macro_Fl - Ratio_Fl * y_Macro_Lu;
y_Macro_OADF = y_Macro_OADF - Ratio_OADF * y_Macro_Lu;
y_Macro_Lu = y_Macro_Lu + (Ratio_Fl + Ratio_OADF) * y_Macro_Lu;

end
```

5 References

1. Cerretani, C.; Palm-Henriksen, G.; Liisberg, M. B.; Vosch, T. The effect of deuterium on the photophysical properties of DNA-stabilized silver nanoclusters. *Chemical Science* **12**, 16100-16105 (2021)
